# Adaptation and Validation of the Malay Version of the Stress and Anxiety to Viral Epidemics-6 Items Scale Among the General Population

**DOI:** 10.3389/fpsyt.2022.908825

**Published:** 2022-06-30

**Authors:** Nicholas Tze Ping Pang, Mathias Wen Leh Tseu, Pradeep Gupta, Jaya Dhaarshini, Assis Kamu, Chong Mun Ho, Oli Ahmed, Seockhoon Chung

**Affiliations:** ^1^Universiti Malaysia Sabah, Kota Kinabalu, Malaysia; ^2^Department of Psychology, University of Chittagong, Chattogram, Bangladesh; ^3^National Centre for Epidemiology and Population Health, Australian National University, Canberra, ACT, Australia; ^4^Department of Psychiatry, Asan Medical Center, University of Ulsan College of Medicine, Seoul, South Korea

**Keywords:** viral epidemic, COVID-19, psychological impact, healthcare, health personnel

## Abstract

The coronavirus disease pandemic has caused untold distress owing to both its physical and psychological sequelae, and such distress is further exacerbated by multiple socioeconomic ramifications. The Stress and Anxiety to Viral Epidemics-6 Items (SAVE-6). Scale was developed to specifically assess the anxiety response of the general population to viral epidemics. This study aimed to establish the psychometric properties of the Malay version of the SAVE-6 Scale in the general population. Herein, a total of 257 individuals participated. World Health Organization instrument validation protocols were used to translate and back-translate the Malay SAVE-6 Scale. Subsequently, the classical test theory and Rasch analysis were used to ascertain the validity and reliability of the scale. Cronbach α was used to measure the internal consistency, which was found to be satisfactory (α = 0.866). The correlations between the SAVE-6 Scale and other scales, including the Generalized Anxiety Disorder-7 Items Scale (r = 0.421, *p* < 0.001) and Patient Health Questionnaire-9 Items (r = 0.354, *p* < 0.001) were significant. Taken together, the Malay version of the SAVE-6 Scale is valid and reliable for use in the general population and is psychometrically suitable for assessing stress and anxiety specific to viral epidemics.

## Introduction

The global population has faced numerous obstacles in the first quarter of the 21^st^ century. Major viral epidemics and pandemics have occurred since 2003. Severe acute respiratory syndrome outbreak emerged in China, which forced the World Health Organization to declare it as a serious global health threat^1^. Thereafter, successive outbreaks, such as the H1N1 influenza (2009), Middle East respiratory syndrome (2012), Ebola outbreak (2013), and coronavirus disease (COVID-19) pandemic (2019), have emerged. COVID-19 was first found in December 2019, and within a span of few weeks, it had spread across the world, prompting the disease to be labeled internationally as a public health emergency by the end of January 2020^2^. The sudden increase in workload and demands for medical equipment, overcrowded health facilities, and many other factors had considerably impacted healthcare systems worldwide.

This phenomenon has caused great degrees of psychopathology in the general population (1–3). Multiple meta-analyses have suggested that the prevalence of depression, anxiety, and stress in the early stages of the pandemic, when knowledge on COVID-19 was still limited, ranged between 29.6 and 33.7% (4). At a later time point, when there was more available knowledge and vaccination strategies had already been implemented on a large scale globally, the prevalence of anxiety remained at around 25% globally, which is thrice higher than that in community settings (5). This prevalence is not merely restricted to adult populations; children and adolescents had a pooled prevalence of depression and anxiety of 25.5%, and crucially, the rates were higher in studies conducted later in the pandemic (6). Given the higher prevalence and sustained presence of multiple psychological pathologies, there is clear evidence that the fear and anxiety related to this pandemic are going to be characterized uniquely. Hence, it is crucial to develop new scales that can measure the specific types of psychological distress that can emerge out of the pandemic.

Malaysia entered its first lockdown on March 18, 2020 after a new cluster was triggered from a mass gathering. Subsequently, Malaysia was in multiple lockdowns until November 2021, when it achieved a herd immunity vaccination rate in the general population^3^. To date, Malaysia has experienced three waves of the COVID-19 pandemic and is currently in the grip of a fourth wave secondary to the Omicron variant, resulting in very high case numbers daily. Fortunately, the high case numbers have not translated into increased morbidity and mortality, as the general population has been universally vaccinated with in excess of 60% of the adult population receiving booster shots as well.

There have been increases in the prevalence of depression and anxiety both among healthcare workers and the general population during the pandemic in Malaysia (7, 8). This has been attributed to fear of the pandemic *per se* and fear secondary to the sequelae of a pandemic (e.g., loss of livelihood, worsened socioeconomic conditions, loss of education and employment opportunities owing to high levels of economic recession and job market shrinkage, and stigma owing to the illness) (9). There have been a few separate scales that can measure the extent of psychological distress; however, these scales are more focused on particular constructs, such as fear and stress (7, 10).

Recently, a new rating scale called the Stress and Anxiety to Viral Epidemics-6 Items (SAVE-6). Scale was developed in response to the need to systematically assess the psychological wellbeing of the general population at any point in response to a viral epidemic (11). This scale was derived from the original SAVE-9 Scale, which was developed to specifically assess work-related stress and viral anxiety of healthcare workers in relation to a viral epidemic (12). The SAVE-6 Scale was validated in different languages among the general population (13–15) and various groups of populations (16–18). The other rating scale, the Malay version of the Fear of COVID-19 scale (10) was validated, and it inquires about an individual's repetitive thoughts or anxiety related physiological arousal symptoms. On the other hand, the SAVE-6 scale inquires about thoughts about the social risk of infection such as the influence on their physical health or about avoidance of others. In this COVID-19 pandemic, assessing one's thought or anxiety on the social effect of COVID-19 is also important.

This study aimed to use both classical test theory (CTT) approach (e.g., factor analysis, internal consistency reliability–Cronbach's alpha, etc.) and modern test theory approaches (e.g., 2PL model, Rasch model, etc.) (19–22) to demonstrate the validity and reliability of the Malay SAVE-6 Scale. CTT is commonly used approach to assess psychometric properties of a test. In this approach, the total scores (the sum of the true score and random error) are utilized for assessing psychometric properties. Here, measurement error is same across the scale. Modern test theory approaches work on response pattern on items by the sample. Therefore, measurement error varies across the scale. Among several models of the modern test theory approach, Rasch model is a utilized to assess psychometric reliability and validity of Likert-type scale. In recent decades, application of this model is increased to assess psychometric properties of a test with the development of modern computer programming. While assessing psychometric properties of a test or scale, both CTT approaches and Rasch model complement each other and provide a detail information about the reliability and validity of a test or scale. Therefore, we utilized both approaches to assess the reliability and validity of the Malay SAVE-6 scale. These are allowing this scale to be used with confidence for assessing psychological wellbeing among the general population during a viral epidemic in the near future and consequently allowing better preparation to address the related psychological health needs.

## Methods

An online survey was conducted on December 1–10, 2021. The validation study was conducted in the general population across both Peninsular and East Malaysia. Recruitment for respondents was performed through convenience snowball method sampling. A Google Form was utilized for data collection to comply with the implementation of strict standard operating procedures on physical distancing and movement control orders. The survey form was developed in Malay and followed the Checklist for Reporting Results of Internet e-Surveys guidelines (23). The usability and technical functionality of the e-survey form were tested by an investigator (N.T.P.P.) before its implementation. All responses were anonymized, and participants could opt out of data collection where indicated. A targeted sample size ranging from 200 to 300 was initially decided, as it is considered a fair-to-good sample size for the purpose of factor analysis (24). Ethical approval was obtained from the Universiti Malaysia Sabah Medical Research Ethics Committee [JKEtika 3/21 (5)] prior to the commencement of this project. All participants provided informed consent.

### Sociodemographic Questionnaire

A simple questionnaire requesting respondents to provide information regarding their working environment and nature, including work position, work duration, healthcare level, and demographic setting, was utilized. While personal identity was kept anonymous, the age range, sex, and marital status were otherwise requested for sociodemographic demonstration purposes. Though Malaysia is a multi-ethnic country, ethnic group information was not collected in this study due to cultural sensitivity issues.

### Psychometric Instruments

#### SAVE-6 Scale

The original SAVE-6 Scale was developed to assess viral anxiety. It consists of six items scored on a five-point Likert scale ranging from 0 (never) to 4 (always) (11). Higher total scores reflect a higher level of viral anxiety. In this study, we applied the translated Malay version (Supplementary Table 1). The SAVE-6 Scale was translated using a back-translation method^4^. Two bilingual experts translated the English version of the SAVE-6 Scale into the Malay version. Thereafter, the two translated Malay versions were synthesized into a single version. The synthesized version was back-translated into English by two other bilingual experts; the two versions were again synthesized into a single version and compared with the original English version to check for any discrepancy in meaning.

#### Generalized Anxiety Disorder-7 Items (GAD-7) Scale

The GAD-7 (25) Scale is a seven-item questionnaire and a widely used self-administered tool for assessing general anxiety. The items are scored on a four-point Likert scale ranging from 0 (not at all) to 3 (nearly every day). The Malay version of the GAD-7 Scale was used in this study (26). The Cronbach α of the GAD-7 Scale was 0.87 among the study sample.

#### Patient Health Questionnaire-9 Items

The PHQ-9 (27) is a self-reported questionnaire consisting of nine items assessing mainly symptoms of depression. Each item is scored on a four-point Likert scale ranging from 0 (not at all) to 3 (nearly every day). The Malay version of the PHQ-9 was used in this study (28). The Cronbach α of the PHQ-9 was 0.96 among the study sample.

### Statistical Analysis

The classical test theory (CTT), item response theory (IRT) and Rasch measurement theory (RMT) were used to check the validity and reliability of the Malay version of the SAVE-6 Scale. For reliability, internal consistency measures using Cronbach α, McDonald's Ω, and greatest lower bound were done. using. Pearson correlation tests comparing the Malay version vs. original English version were used. For validity, convergent validity (vs. Malay GAD-7 Scale and Malay PHQ-9) was used. The dimensionality of the Malay SAVE-6 Scale was explored using exploratory factor analysis, which uses principal axis factor as the extraction method and promax as the rotation method. The effectiveness of the Malay SAVE-6 Scale as a diagnostic instrument for assessing stress and anxiety and the appropriate cut-off point of the total score were determined using receiver operating characteristic (ROC) curve analysis. Finally, the Kruskal–Wallis test was conducted to compare the Malay SAVE-6 Scale scores according to the groups of participants classified on the basis of the Malay GAD-7 Scale and Malay PHQ-9 scores. The analysis was conducted using IBM SPSS 26.0 and JAPS 0.16.

For IRT, we run the graded response model (GRM) that suitable for likert type response option. Before running the GRM, IRT assumptions {unidimensionality [Loevinger's H coefficient], local dependance [p values (adjusted for false discovery rate) of G^2^], and monotonicity [number of significant violations and *Crit* value]} were assessed to examine the suitability for IRT. Next, item fits were assessed through S-χ^2^ and its *p* values [adjusted for false discovery rate]. In GRM, there are two parameters in-slope/ discrimination parameter (α) and threshold/ difficulty parameters (*b*) of items. Both parameters in GRM, local dependence and item fits were estimated using the R package version *mirt* version 1.34. Unidimensionality and monotonicity were estimated through R package *mokkoen* version 3.0.6.

For the RMT, weighted fit statistics (infit) and outlier sensitive fit statistic (outfit) mean square (MnSq) values were used at the item level, while item and person separation reliability values and item and person separation indices were applied at the scale level. MnSq values close to 1 suggest a good model-data fit. The accepted range of the infit and outfit MnSq values is between 0.5 and 1.5 (29, 30). The recommended item and person reliability values are 0.7 or higher (31), while the recommended item and person separation indices are 2 or higher (32). The Rasch analysis was conducted using jMetrik 4.1.1.

## Results

A total of 257 individuals from the general population participated in the survey; of them, 63.8% were women; 77.8% were single; and 10.9% experienced being quarantined. Approximately 52.5% responded that they experienced past psychiatric symptoms, and 29.6% responded that they were currently experiencing psychiatric symptoms at the time of the survey (Table 1).

**Table 1:** Respondents' background information (*n* = 257).

		***N***	**%**
Age group (year)	<20	37	14.4%
	20–29	156	60.7%
	30–39	34	13.2%
	40–49	21	8.2%
	≥50	9	3.5%
Sex	Female	164	63.8%
	Male	93	36.2%
Marital status	Single	200	77.8%
	Married	57	22.2%
Have you ever been infected with coronavirus disease and underwent a quarantine process?	Yes	28	10.9%
Have you ever experienced or been treated for depression, anxiety, or insomnia?	Yes	135	52.5%
Do you feel that you are experiencing depression or anxiety or need help dealing with your current emotions/moods?	Yes	76	29.6%

### Dimensionality of the Malay SAVE-6 Scale

The Kaiser–Meyer–Olkin measure verified the sampling adequacy for the factor analysis, as the value was more than 0.5 (0.878). Bartlett's test of sphericity [*X*^2^_15_ = 673.224, *p* < 0.001] also confirmed that relationships existed between at least some of the six items, indicating that the correlation structure was adequate for the factor analysis. The principal axis factor confirmed the unidimensionality of the Malay SAVE-6 Scale, as there was only one factor extracted. The eigenvalue for the factor was 3.185. The factor could explain 53.1% of the variation in the six items. The factor loadings and Cronbach α are shown in Table 2.

**Table 2:** Descriptive statistics of the Malay version of the Stress and Anxiety to Viral Epidemics-6 Items scale (*n* = 257).

**Item**	**Min**	**Max**	**Mean ±standard deviation**	**Skewness**	**Kurtosis**	**Coefficient of variation**	**Factor loading**
Item 1	0.00	4.00	2.26 ± 1.02	−0.099	−0.126	0.448	0.749
Item 2	0.00	4.00	2.27 ± 1.11	−0.117	−0.553	0.490	0.787
Item 3	0.00	4.00	2.57 ± 1.01	−0.118	−0.586	0.392	0.823
Item 4	0.00	4.00	2.35 ± 1.09	−0.213	−0.533	0.463	0.723
Item 5	0.00	4.00	1.61 ± 1.16	0.106	−0.729	0.717	0.568
Item 6	0.00	4.00	2.83 ± 1.08	−0.742	0.056	0.382	0.694

### Reliability and Validity of the Malay SAVE-6 Scale

All psychometric measurements are shown in Table 3. The internal consistency measures, including Cronbach's α (0.866) and McDonald's (0.866), confirmed the validity and reliability of the Malay SAVE-6 Scale, as all values passed the suggested cut-off points.

**Table 3:** Psychometric properties of the Malay version of the SAVE-6 scale at the scale level (*n* = 257).

**Psychometric measure**	**Result**	**Suggested cut-off**
Internal consistency measure using Cronbach α	0.866	>0.7
Internal consistency measure using McDonald's Ω	0.866	>0.7
Internal consistency measure using the greatest lower bound	0.897	>0.7
Test-retest reliability (Malay and original versions)	0.849^**^	See Note
Convergent validity (SAVE-6 scale vs. Malay GAD-7 scale)	0.421^**^	See Note
Convergent validity (SAVE-6 scale vs. Malay PHQ-9)	0.354^**^	See Note

** *The correlation is significant at the 0.01 level (two-tailed test). Correlation coefficients of <0.25 are considered as small; 0.25–0.50, moderate; 0.50–0.75, good; and >0.75, excellent. SAVE-6, Stress and Anxiety to Viral Epidemics-6 Items*.

### Cut-Off Score for the Malay SAVE-6 Scale

The ROC graph displays a convex pattern indicating a good discrimination ability. The area under the curve (AUC) demonstrated a solid diagnostic accuracy [AUC value = 0.729 (95% confidence interval = 0.661–0.797); *p* < 0.001]. The appropriate cut-off score was determined as 13.5–14, with good sensitivity (0.605) and specificity (0.756).

### Malay SAVE-6 Scale Scores Based on the Anxiety and Depression Levels

The Kruskal–Wallis test showed that the total scores for the Malay SAVE-6 Scale were significantly different among the three groups based on the anxiety levels (Malay GAD-7 Scale score of 0, 1–4, and ≥5) and depression levels (Malay PHQ-9 score of 0, 1–9, and ≥10) (Supplementary Table 2).

### Graded Response Model Results

**Figure 1:**
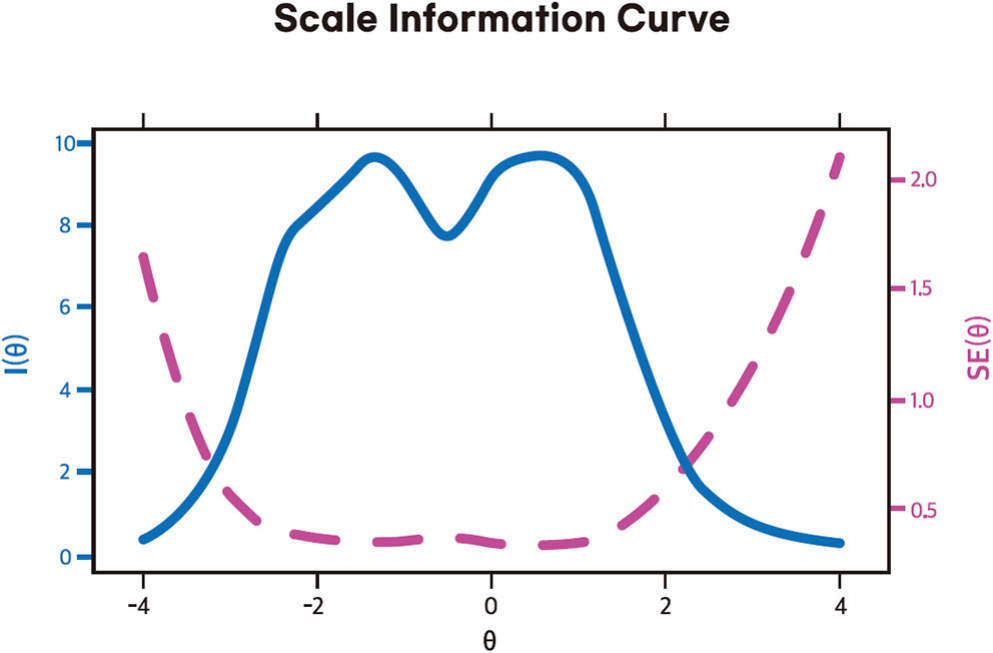
Scale information curve of the Malay version of the SAVE-6 scale among the general population.

Results about IRT assumptions are presented in Supplementary Table 3. Loevinger's H coefficient value (0.575) suggested this scale as highly unidimensional. Non-significant G^2^ *p*-values (adjusted for false recovery rate) suggested absence of possible local dependance between items. Supplementary Table 3 also shows that the number of both significant violation and crit value for each item are 0. These results suggested absence of monotonicity of items. Overall, IRT assumptions meet to run an IRT model to assess psychometric properties of the scale. Supplementary Table 4 shows the GRM outputs. Non-significant *p*-values of S-χ^2^ suggested all items belong to same scale (SAVE-6 Malay version). About slope parameter, all items have very high slope parameters except item 5 that has high slope parameters (range: 1.368–3.397). These high and very high slope parameters suggested that these items provide good information about the latent trait that assessed by this scale. About threshold parameter, item 6 is least difficult compared to the rest of the items. Higher latent trait or theta is required to endorse Likert-type response options “often” and “always” in items 1-5. In item 6, higher latent trait or theta is required to endorse Likert-type response option “always.” Figure 1 present the scale information curve of the SAVE-6 Malay version. Scale information curve shows that the SAVE-6 Malay version efficient to assess the latent trait between−3.25 and 2.25 theta level.

### RMT Results

The quality of the Malay SAVE-6 Scale was also satisfactory, as all item and person reliability values and indices exceeded the suggested cut-off points (Supplementary Table 5). The quality of the related scales (Malay GAD-7 Scale and Malay PHQ-9) was also shown. The item and person reliability values and indices indicated that all related scales also met the minimum quality requirement based on the Rasch model (Supplementary Table 6).

## Discussion

Our validation study is a crucial validation process, as it allows the measurement of general mental health wellbeing among the general population using a scale that has been demonstrated to have reasonably good psychometric properties. The Malay SAVE-6 Scale fulfills all these criteria, demonstrating reasonable test-retest reliability, convergent validity to two separate measures, and internal consistency using three separate measures. Our findings show that there are two distinct factors that are distinguishable from each other, which corroborates the original SAVE-6 Scale factor structure, and there is an identifiable cut-off point on the ROC curves, with acceptable sensitivity and specificity. The Malay SAVE-6 Scale is valid and reliable based on the results of the psychometric analysis: Its internal consistency was confirmed by Cronbach α (0.866) and McDonald's Ω (0.866), whereas its validity was confirmed by the significant convergent validity with the PHQ-9 and GAD-7 Scale. Hence, this scale is a valuable addition to and supplements other pandemic-specific scales that have been developed for Malaysian (7, 10) and regional (33) use.

The factor loading of item 5 (Are you worried that others might avoid you even after the infection risk has been minimized?) was relatively low (0.568) among the study sample. This low value of the SAVE-6 was reported in the previous study among the general population (15) and healthcare workers (34). A possible explanation is that, first, the general population has adjusted to the long period of the pandemic. In addition, stigmatization is no longer a serious problem, since many individuals and their neighbors have already been infected. Second, the usefulness of item 5 may be related work-related stress of healthcare workers rather than viral anxiety of the general population. Originally, the SAVE-9 scale was clustered into two factors: pandemic-related anxiety (item 1, 2, 3, 4, 5, and 8) and work-related stress (item 6, 7, and 9) (12). However, from the results of studies conducted among healthcare workers in Russia (35) and German (36), the item 5 was clustered into factor of work-related stress. Although we need to consider the cultural differences, we may consider whether the item 5 is excluded or not from the SAVE-6 in the further study.

The cut-off score for the Malay SAVE-6 Scale in this study was 14 based on the mild degree in the GAD-7 scale. The SAVE-6 scale was originally developed to identify individuals who need psychological support, and a rating scale that can measure at least mild degrees of generalized anxiety during this pandemic has been attempted to be developed. Previously, a cut-off score of 15 among the general population or medical students in Korea (11), 16 among public workers in Korea (18), and 12 among the general population in Lebanon (13) have been reported. The cut-off score of 14 for the SAVE-6 scale among the general population in Malaysia might have been influenced by the difference in the COVID-19 situation or culture. This is reflective of the burgeoning evidence suggesting that there are higher levels of anxiety specific to the COVID-19 pandemic across various large multinational studies (8, 9, 37). This anxiety can be divided into various stages. At the early stage of the pandemic, it was more reflective of the fear of death and high levels of uncertainty engendered by a rapidly evolving pandemic. As lockdown after interminable lockdown ensued, anxiety increasingly stemmed from lost educational, economic, and relationship opportunities and feelings of isolation, loneliness, and detachment from the regular processes of society. As the second year of the pandemic began, further anxieties were fueled by the fact that the promised end of the pandemic that was initially guaranteed by vaccinations was not in sight. This was further exacerbated by the rise of the highly contagious Delta and Omicron variants^5^, increasing infection rates in fully vaccinated populations (38) and resulting in booster mandates being rolled out in various countries. Hence, it is crucial that the SAVE-6 scale is developed in the Malay language, as Malaysia continues to experience moderately high levels of reinfection and breakthrough infection despite a high vaccination rate (39).

A few limitations inherently exist in cross-sectional projects, including our study, which statistics attempt to mitigate. First, the participants were recruited via an online survey. This might influence the accessibility of participants, and educated individuals with internet-enabled and digital devices could easily access the survey, which may lead to bias. Second, strict national lockdowns and social distancing protocols necessitated fully online data collection protocols, which are potential restrictions on participant reach. Furthermore, small sample size of this study might lead to bias. Second, convenience snowball sampling was the methodology utilized to collect data; however, this might impact how representative the targeted population is. Third, the study used a self-rated survey, which might be subject to certain biases, such as social desirability bias. Last, the validity of the Malay version of the SAVE-6 was not explored among different ethnicity in Malaysia, a multi-ethnic country. We did not collect the group information in this study as considering cultural sensitivity issues.

In conclusion, the Malay version of the SAVE-6 scale has high levels of validity and reliability in both the CTT and Rasch analysis, with reasonable cut-off points in the ROC curve analysis and fair sensitivity and specificity. Thus, it is a valuable addition to pandemic-sensitive tools measuring anxiety and fear. Considering the validated cut-off points, the levels of detection of psychopathology in the general population will increase, allowing more efficacious interventions, such as cognitive behavioral therapy or mindfulness-based therapy, to be initiated earlier to achieve maximal benefits.

## Data Availability Statement

The raw data supporting the conclusions of this article will be made available by the authors, without undue reservation.

## Ethics Statement

Ethical approval was obtained from the Universiti Malaysia Sabah Medical Research Ethics Committee [JKEtika 3/21 (5)] prior to the commencement of this project. All participants provided informed consent. The patients/participants provided their written informed consent to participate in this study.

## Author Contributions

NP and SC: conceptualization. AK and OA: formal analysis and methodology. NP, SC, MT, PG, JD, AK, and CH: data curation and writing—original draft. OA: visualization. All authors: writing—review and editing, contributed to the article, and approved the submitted version.

## Conflict of Interest

The authors declare that the research was conducted in the absence of any commercial or financial relationships that could be construed as a potential conflict of interest.

## Publisher's Note

All claims expressed in this article are solely those of the authors and do not necessarily represent those of their affiliated organizations, or those of the publisher, the editors and the reviewers. Any product that may be evaluated in this article, or claim that may be made by its manufacturer, is not guaranteed or endorsed by the publisher.

## Footnotes

^1^Available online at: https://cdn.who.int/media/docs/default-source/documents/health-topics/mers/wha56-29-agenda-item-14-16-sars.pdf?sfvrsn=16254555_8 (accessed March 30, 2022).

^2^Available online at: https://www.who.int/director-general/speeches/detail/who-director-general-s-statement-on-ihr-emergency-committee-on-novel-coronavirus-(2019-ncov) (accessed March 30, 2022).

^3^Available online at: https://www.nst.com.my/news/nation/2022/01/759449/64-omicron-variant-cases-detected-malaysia (accessed March 30, 2022).

^4^Available online at: http://www.who.int/substance_abuse/research_tools/translation/en/ (accessed March 10, 2022).

^5^Available online at: https://www.nst.com.my/news/nation/2022/01/759449/64-omicron-variant-cases-detected-malaysia (accessed March 30, 2022).

## Supplementary Material

Supplementary Table 1 Original english and malay versions of the stress and anxiety to viral epidemics-6 items scale.

Supplementary Table 2 Malay SAVE-6 scale scores among the groups classified according to the GAD-7 scale and PHQ-9 scores.

Supplementary Table 3 Loevinger's H coefficient, monotonicity, and G2 p values of items of the Malay version of the SAVE-6 scale.

Supplementary Table 4 GRM output.

Supplementary Table 5 Scale quality statistics based on the rasch model (n = 257).

Supplementary Table 6 Item statistics of the malay version of the stress and anxiety to viral epidemics-6 items scale based on the rasch model (n = 257).
