# Supplementary material for: Adaptation and Validation of the Malay Version of the Stress and Anxiety to Viral Epidemics-6 Items Scale Among the General Population
Source: Front Psychiatry. 2022 Jun 30;13:908825. doi: 10.3389/fpsyt.2022.908825 (PMC9279658; doi:10.3389/fpsyt.2022.908825)
Supplement: Supplementary Table 1 — Original english and malay versions of the stress and anxiety to viral epidemics-6 items scale. [file Data_Sheet_1.docx]

**Supplementary Table 1. Original English and Malay versions of the Stress and Anxiety to Viral Epidemics-6 Items Scale**

| **Item** | **Original English version** | **Malay version** |
| --- | --- | --- |
| **Item 1** | Are you afraid the virus outbreak will continue indefinitely? | *Adakah anda takut wabak virus akan berterusan selama-lamanya?* |
| **Item 2** | Are you afraid your health will worsen because of the virus? | *Adakah anda takut kesihatan anda akan bertambah teruk kerana virus?* |
| **Item 3** | Are you worried that you might get infected? | *Adakah anda bimbang anda mungkin dijangkiti?* |
| **Item 4** | Are you more sensitive towards minor physical symptoms than usual? | *Adakah anda lebih peka terhadap gejala fizikal yang kecil daripada biasa?* |
| **Item 5** | Are you worried that others might avoid you even after the infection risk has been minimized? | *Adakah anda bimbang bahawa orang lain mungkin mengelakkan anda, walaupun selepas risiko jangkitan telah berkurang?* |
| **Item 6** | Do you worry your family or friends may become infected because of you? | *Adakah anda bimbang keluarga atau rakan anda mungkin dijangkiti disebabkan anda?* |

**Supplementary Table 2. Malay SAVE-6 scale scores among the groups classified according to the GAD-7 scale and PHQ-9 scores**

|  | Total score | N | SAVE-6 score (Mean ± SD) | Kruskal–Wallis test |
| --- | --- | --- | --- | --- |
| **GAD-7 scale** | 0 | 30 | 7.60 ± 4.55 | H=33.864,  *p*<0.001 |
|  | 1–4 | 72 | 10.79 ± 4.07 |  |
|  | ≥5 | 155 | 12.81 ± 3.70 |  |
| **PHQ-9 scale** | 0 | 17 | 7.88 ± 4.85 | H=26.3688,  *p*<0.001 |
|  | 1–9 | 156 | 11.14 ± 4.04 |  |
|  | ≥10 | 84 | 13.31 ± 3.83 |  |

These item fit statistics show that each item of the Malay SAVE-6 scale meets the unidimensional requirement of a Rasch model, as all the values are within 0.5–1.5. The most difficult item was item 5, and the easiest item was item 3. SAVE-6, Stress and Anxiety to Viral Epidemics-6 Items; GAD-7, Generalized Anxiety Disorder-7 Items; PHQ-9, Patient Health Questionnaire-9 Items

**Supplementary Table 3. Loevinger’s H coefficient, monotonicity, and G^2^ p values of items of the Malay version of the SAVE-6 scale.**

|  | ***H* coefficients** | **Monotonicity** | | | | **Local dependance G^2^ p values** | | | | |
| --- | --- | --- | --- | --- | --- | --- | --- | --- | --- | --- |
|  |  | **#ac** | **#vi** | **#zsig** | ***Crit*** | **Item1** | **Item2** | **Item3** | **Item4** | **Item5** |
| **Item1** | .70 | 19 | 0 | 0 | 0 |  |  |  |  |  |
| **Item2** | .74 | 16 | 0 | 0 | 0 | .386 |  |  |  |  |
| **Item3** | .74 | 5 | 0 | 0 | 0 | .186 | .181 |  |  |  |
| **Item4** | .62 | 19 | 0 | 0 | 0 | .737 | .181 | .186 |  |  |
| **Item5** | .58 | 21 | 0 | 0 | 0 | .255 | .181 | .197 | .435 |  |
| **Item6** | .65 | 21 | 0 | 0 | 0 | .186 | .181 | .181 | .313 | .120 |
| ac = active comparison, vi = violation, zsig = significant violation  Notes: p-values adjusted for false discovery rate (FDR) | | | | | | | | | | |

**Supplementary Table 4. GRM output**

| **Items** | **Item fits** | | | **Slope parameter (a)** | **Threshold parameter (b)** | | | |
| --- | --- | --- | --- | --- | --- | --- | --- | --- |
|  | **S-χ^2^** | **df** | **p value** |  | **b_1_** | **b_2_** | **b_3_** | **b_4_** |
| **Item 1** | 19.389 | 20 | .596 | 2.504 | -1.950 | -1.125 | .429 | 1.350 |
| **Item 2** | 24.074 | 24 | .596 | 2.752 | -1.770 | -.908 | .313 | 1.168 |
| **Item 3** | 25.733 | 16 | .116 | 3.397 | -2.421 | -1.275 | .040 | .851 |
| **Item 4** | 21.584 | 25 | .660 | 1.959 | -2.184 | -1.088 | .190 | 1.273 |
| **Item 5** | 54.273 | 31 | .036 | 1.368 | -1.185 | -.412 | 1.289 | 2.518 |
| **Item 6** | 41.023 | 26 | .093 | 2.031 | -2.295 | -1.676 | -.488 | .573 |
| Notes: p-values adjusted for false discovery rate (FDR) | | | | | | | | |

**Supplementary Table 5. Scale quality statistics based on the Rasch model (n=257)**

| **Psychometric measure** | **Malay SAVE-6** | **Malay GAD-7** | **Malay PHQ-9** | **Suggested cut-off point** |
| --- | --- | --- | --- | --- |
| **Item separation reliability value** | 0.9873 | 0.9722 | 0.9782 | ≥0.7 |
| **Item separation index** | 8.8256 | 5.9155 | 6.6925 | ≥2 |
| **Person separation reliability value** | 0.8591 | 0.8969 | 0.8540 | ≥0.7 |
| **Person separation index** | 2.4695 | 2.9502 | 2.4183 | ≥2 |

SAVE-6, Stress and Anxiety to Viral Epidemics-6 Items; GAD-7, Generalized Anxiety Disorder-7 Items; PHQ-9, Patient Health Questionnaire-9 Items

**Supplementary Table 6. Item statistics of the Malay version of the Stress and Anxiety to Viral Epidemics-6 Items scale based on the Rasch model (n=257)**

| **Item** | **Infit MnSq** | **Outfit MnSq** | **Difficulty** |
| --- | --- | --- | --- |
| **Item 1** | 0.94 | 0.95 | 0.13 |
| **Item 2** | 0.86 | 0.83 | 0.15 |
| **Item 3** | 0.78 | 0.80 | -0.98 |
| **Item 4** | 0.99 | 1.01 | -0.03 |
| **Item 5** | 1.36 | 1.39 | 1.55 |
| **Item 6** | 1.06 | 1.03 | -0.83 |

MnSq, mean square error
